# The Ran GTPase-Activating Protein (RanGAP1) Is Critically Involved in Smooth Muscle Cell Differentiation, Proliferation and Migration following Vascular Injury: Implications for Neointima Formation and Restenosis

**DOI:** 10.1371/journal.pone.0101519

**Published:** 2014-07-02

**Authors:** Marc Vorpahl, Sabine Schönhofer-Merl, Cornelia Michaelis, Annette Flotho, Frauke Melchior, Rainer Wessely

**Affiliations:** 1 Department of Cardiology, HELIOS Klinikum Wuppertal, University Witten/Herdecke, Witten, Germany; 2 Deutsches Herzzentrum Muenchen and 1. Medizinische Klinik, Klinikum rechts der Isar, Technische Universitaet Muenchen, Muenchen, Germany; 3 Zentrum für Molekulare Biologie der Universität Heidelberg (ZMBH), DKFZ - ZMBH Alliance, Heidelberg, Germany; 4 Zentrum fuer Herz-Gefaess-Lungenmedizin, Cologne, Germany; SUNY College of Nanoscale Science and Engineering, United States of America

## Abstract

Differentiation and dedifferentiation, accompanied by proliferation play a pivotal role for the phenotypic development of vascular proliferative diseases (VPD), such as restenosis. Increasing evidence points to an essential role of regulated nucleoporin expression in the choice between differentiation and proliferation. However, whether components of the Ran GTPase cycle, which is of pivotal importance for both nucleocytoplasmic transport and for mitotic progression, are subject to similar regulation in VPD is currently unknown. Here, we show that differentiation of human coronary artery smooth muscle cell (CASMC) to a contractile phenotype by stepwise serum depletion leads to significant reduction of RanGAP1 protein levels. The inverse event, dedifferentiation of cells, was assessed in the rat carotid artery balloon injury model, a well-accepted model for neointima formation and restenosis. As revealed by temporospatial analysis of RanGAP1 expression, neointima formation in rat carotid arteries was associated with a significant upregulation of RanGAP1 expression at 3 and 7 days after balloon injury. Of note, neointimal cells located at the luminal surface revealed persistent RanGAP1 expression, as opposed to cells in deeper layers of the neointima where RanGAP1 expression was less or not detectable at all. To gain first evidence for a direct influence of RanGAP1 levels on differentiation, we reduced RanGAP1 in human coronary artery smooth muscle cells by siRNA. Indeed, downregulation of the essential RanGAP1 protein by 50% induced a differentiated, spindle-like smooth muscle cell phenotype, accompanied by an upregulation of the differentiation marker desmin. Reduction of RanGAP1 levels also resulted in a reduction of mitogen induced cellular migration and proliferation as well as a significant upregulation of the cyclin-dependent kinase inhibitor p27^KIP1^, without evidence for cellular necrosis. These findings suggest that RanGAP1 plays a critical role in smooth muscle cell differentiation, migration and proliferation *in vitro* and *in vivo*. Appropriate modulation of RanGAP1 expression may thus be a strategy to modulate VPD development such as restenosis.

## Introduction

Vascular proliferative diseases such as in-stent restenosis, bypass atherosclerosis and transplant vasculopathy are of critical clinical importance, leading to a significant morbidity and mortality worldwide [1]–[3]. Remodeling processes like cellular proliferation and migration, in particular of vascular smooth muscle cells (SMC), have been shown to significantly contribute to the pathogenesis of these diseases [5]–[6].

Increasing evidence suggests a critical role of the nucleocytoplasmic transport machinery for cell differentiation as well as co-regulation of cellular mitosis. Exchange processes between the nucleus and the cytoplasm are accommodated via nuclear pore complexes (NPCs), macromolecular machines that allow passive diffusion of ions and metabolites but serve as a diffusion barrier for most macromolecules [7], [8]. Active transport of proteins and ribonucleoprotein particles across NPCs is hence an essential process in all eukaryotic cells. It is mediated by soluble transport receptors (importins and exportins) that recognize nuclear import or export signals and their respective cargo molecules and carry them through nuclear pore complexes. Assembly and disassembly of transport complexes is controlled by the small GTPase Ran and its essential auxiliary factors, the guanine nucleotide exchange factor RCC1 (regulator of chromosome condensation 1) and the Ran GTPase activating protein RanGAP1. Their asymmetric intracellular localisation - RCC1 is restricted to the nucleoplasm and RanGAP1 is exclusively cytoplasmic - is crucial for directional nucleocytoplasmatic transport. A significant fraction of RanGAP1 is anchored to cytoplasmic filaments of the NPC, by virtue of modification with the small ubiquitin-related modifier SUMO1 and subsequent complex formation with the nucleoporin Nup358/RanBP2 [9]–[11].

Several recent studies indicate that changes in nuclear pore complex composition contribute to cell differentiation. For example, induced expression of the integral transmembrane nucleoporin Nup210 is essential for differentiation of myoblasts into myotubes [12]. Moreover, increased incorporation of the peripheral cytoplasmic nucleoporin Nup358/RanBP2 correlates with structural changes of NPCs and increased nuclear export rates in myotubes compared to myoblasts [13]. In addition to NPC components, soluble factors of the nucleocytoplasmic transport machinery have been reported to be important during differentiation. For example, specific importin alpha paralogs have been shown to influence myoblast proliferation, myotube growth, and myocyte migration [14], and mutations in Drosophila RCC1 at the zygotic stage alter neural differentiation [15]. Specific regulation of RanGAP1, however, has so far not been described.

The aim of the study was to investigate whether RanGAP1 levels change during differentiation of human coronary artery smooth muscle cells (CASMC), to examine the impact of RanGAP1 on differentiation, proliferation and migration of CASMC by sequence specific posttranscriptional gene silencing with small interfering RNA (siRNA) molecules, and to assess its *in vivo* importance by assessment of its regulation in an established animal model of neointima formation, the rat carotid artery balloon-injury model.

## Methods

### Cell culture

Human coronary artery smooth muscle cells (CASMC, #CC-2583; Clonetics, Walkersville, MD) were obtained at passage 3 and used in passages not higher than 10. CASMC were grown in smooth muscle cell basal medium (SmBM, #CC-3182; Clonetics, Walkersville, MD), including 0.5 µg/ml hEGF, 5 mg/ml insulin, 1 µg/ml bFGF (basic fibroblast growth factor), 50 mg/ml gentamicin and 5% FBS; (#CC-4149; Clonetics, Walkersville, MD) at 37°C in 5% CO_2_. A differentiated CASMC phenotype was induced by serum depletion [4] by smooth muscle cell basal medium (SmBM, #CC-3182; Clonetics, Walkersville, MD), including 0.5 µg/ml hEGF (human epidermal growth factor), 5 mg/ml insulin, 1 µg/ml bFGF and 50 mg/ml gentamicin w/o FBS (fetal bovine serum) at 37°C in 5% CO_2_ for 12 h, 24 h, 36 h, 48 h, 60 h and 72 h.

### siRNA Design, Synthesis, and Labeling

siRNAs were designed after determination of target sequences by aligning the RanGAP1 sequence to an Ambion (Grand Island, NY) Web-based algorithm (http://www.ambion.com/techlib/misc/siRNA_finder.html). siRNA duplex oligonucleotides were manufactured by Dharmacon (Lafayette, CO) in the “ready-to-use” option. The 21-nucleotide duplex siRNA molecules with 3-dTdT overhangs were resuspended in nuclease-free water according to the instructions of the manufacturer (Table 1). To ensure stringent controls, a scrambled control sequence (siRNA-SCR) obtained from Ambion (Silencer Negative Control No. 1 siRNA, catalogue No. 4610) was used. To study the distribution pattern of siRNA in cell culture, duplex siRNAs were fluorescence labelled with the use of the Silencer Cy3 (indocarbocyanin) RNA Labeling Kit (Ambion, #1632) according to the protocol provided by the manufacturer.

**Table 1:** siRNA Sequences.

Name	5-3 Sequence	Target gene
RanGAP1-1	GACCUUGCGGCAGGUGGAG dTdT	RanGAP1
RanGAP1-2	UGGCAACACCCUGGAGAA dTdT	RanGAP1
siRNA-SCR	n/a	none

All molecules consist additionally of dTdT overhangs. n/a indicates not avaible (commercially available scrambled siRNA obtained from Ambion).

### Transfection of siRNA

For siRNA transfection, cells were grown to 30–40% confluence in 6-well plates (Falcon/Becton-Dickinson, Heidelberg, Germany) and transfected with the use of 4 µL Oligofectamine reagent (Invitrogen, Carlsbad, CA; #12252-011), 150 pmol siRNA RanGAP1-1 and 150 pmol siRNA RanGAP1-2 and OptiMEM medium (Invitrogen, #31985-047) up to a final volume of 1 mL. Transfection mixtures were left on cells for 4 h. After washing, cells were incubated with supplemented medium for 48 h.

### Assessment of Cell Proliferation and Viability (LDH Assay)

Cell proliferation was assessed by counting cells in random x100-power fields (≥3 fields per chamber) by means of an Axiovert 10 inverted microscope (Zeiss, Jena, Germany). Cytotoxicity was assessed by determination of LDH release from the cytosol of injured cells into the supernatant. LDH was quantified by a colorimetric assay (Roche, Mannheim, Germany, No. 1644793) as previously described [16]. Maximum LDH release was determined by 1% (vol/vol) TritonX-100 (Sigma, Munich, Germany #X-100) treatment.

### Migration assay

Influence of RanGAP1 siRNA on smooth muscle cell migration was measured in a Boyden chamber system. The QCMi-FN quantitative cell migration assay (#ECM500) from Chemicon (Temecula, CA) was applied according to the protocol of the manufacturer. This assay allows measurement of cell migration (haptotaxis) towards a fibronectin gradient [16]. As controls, BSA (bovine serum albumin) coated chambers were used. After 18 h, cells on the bottom side of the membrane were fixed, stained with crystal violet and manually counted on an inverted microscope.

### Western blotting

Western blotting was performed as described previously [16]. Membranes were probed with antibodies directed against p27^KIP1^ (BD Transduction labs, Lexington, KY; #610241), alpha smooth muscle-actin (Abcam, Cambridge, United Kingdom, #ab5694), desmin (#ab32362), actin (Santa Cruz Biotechnology Inc, Santa Cruz, CA; SC#1616) and goat anti-RanGAP1 antibody [17]. Western blot bands were quantified using QuantityOne software (Bio-Rad Laboratories, Munich, Germany) by measuring the band intensity (Area×OD) for each group and normalizing to α-actin. The final results are expressed as percent changes by normalizing the data to the control values.

### Rat carotid injury model

To determine the regulation of RanGAP1 during neointima formation, an established animal model of restenosis was used, namely the rat carotid injury model. At first, the left common carotid artery was exposed and injured by withdrawal of an inflated 2 French Fogarty catheter as described previously [18]. 3 days, 7 days and 14 days after surgery, animals were sacrificed under isoflurane anaesthesia and the common carotid artery was embedded in paraffin as described previously [19]. All animal work was conducted in accordance with German Federal Animal Protection Laws and approved by the Institutional Animal Care and Use Committee at the Technical University of Munich.

For immunohistological analysis, 5 µm thick sections were cut with a rotation microtome (Microm GmbH, Walldorf, Germany). The slices were stained with haematoxylin-eosin and anti-RanGAP1 antibody. Omitting the primary antibody controlled for non-specific binding of antibodies. Computer-assisted morphometric analysis was performed on high-resolution images of the cross sections (IPLab, BD Bioscience Bioimaging, Rockville, MD).

### Statistical analysis

Results are expressed as mean ± SD. The significance of variability among the means of the experimental groups was determined by 1- or 2-way ANOVA. All statistical tests were performed by using the software JMP (Version 7. SAS Institute Inc., Cary, NC, 1989–2007). Differences among experimental groups were considered statistically significant at *P*<0.05.

## Results

### RanGAP1 protein levels are reduced as CASMC enter quiescence

**Figure 1:**
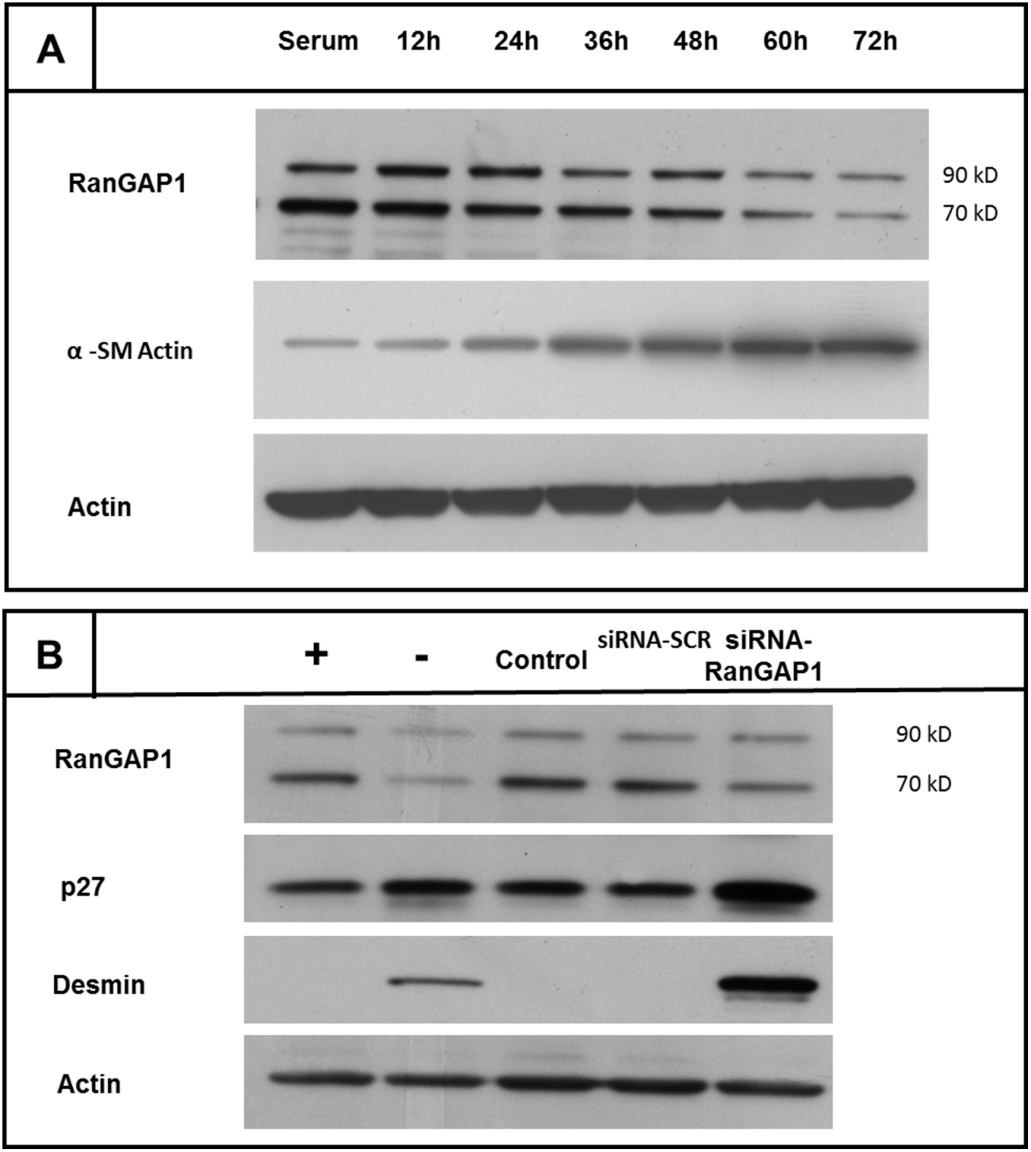
Downregulation of RanGAP1 in arrested, differentiated smooth muscle cells (A). To induce differentiation, CASMCs were depleted from serum. RanGAP1 expression in CASMC was assessed by Western Blotting 12(∼70 kDa) form as well as the SUMO-1 conjugated (∼90 kDa) form of the RanGAP-1 protein. Both bands revealed reduced RanGAP1 expression over time in cells entering quiescence. α-SM Actin protein, a marker of cell differentiation, is increased over time. Actin levels are displayed as loading control. **Effect of posttranscriptional gene silencing of RanGAP1 by small interfering RNA (siRNA) on cell cycle and differentiation markers (B).** siRNA mediated gene silencing of RanGAP1 was able to reduce the 90 kD band by 31.8±21.2% (90 kD band) and 75%±14.7% (70 kD band) 48 h post transfection, respectively (average of three different experiments). RanGAP1 depletion was associated with a strong increase of p27^Kip1^ expression by 60±34%. RanGAP1 deficiency was also associated with a sharp increase in desmin expression with levels even higher than in quiescent cells. CASMC denotes coronary artery smooth muscle cells; (+) denotes serum stimulated CASMC; (−) denotes quiescent CASMC (≥72 h serum depletion); “control” denotes oligofectamine transfected cells without siRNAs; siRNA-SCR denotes scrambled (control) siRNA.

To test whether RanGAP1 levels are subjected to regulation in response to cell differentiation, we induced a differentiated CASMC phenotype by serum depletion for 12 h, 24 h, 36 h, 48 h, 60 h and 72 h. RanGAP1 expression was examined by immunoblotting. As expected, two immunoreactive bands were detected, one for unmodified RanGAP1 at 70 kD and one for sumoylated RanGAP1 at 90 kD. Serum depletion induced an incremental downregulation of RanGAP1 over time (90 kD band: −43.8±19.4%; 70 kD band: −76.2±8%). Concurrently, α-SM actin, a marker of cell differentiation, increased over time (Figure 1A).

### siRNA mediated reduction of RanGAP1 induces a differentiation-related phenotype in CASMC

Reduction of RanGAP1 protein could be a consequence of - but may also contribute to differentiation. To gain first insights into this question, we employed siRNA mediated knockdown.

**Figure 2:**
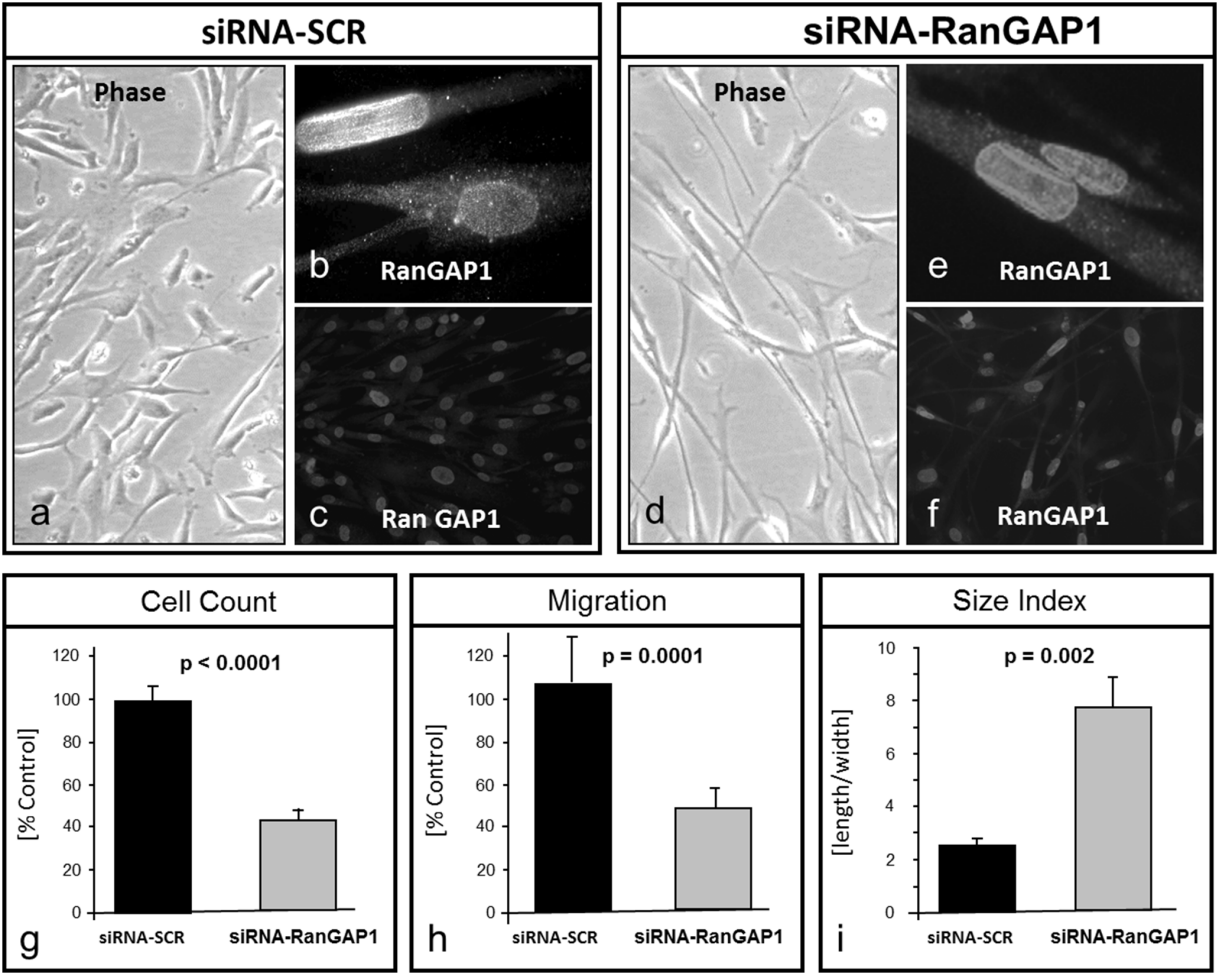
Gene silencing of RanGAP1 by siRNA in CASMC. CASMC fixed and permeabilized with 4% PFA and 0.2% Triton X-100 were subjected to indirect immunofluorescence with an RanGAP1 antibody. CASMC treated with control scrambled siRNA reveal accumulation of RanGAP1 expression at the nuclear rim rather than in the cytosol (a–c). Likewise, siRNA-RanGAP1 mediated gene silenced CASMC show a residual RanGAP1 expression mainly at the nuclear rim (d–f). Gene silencing of RanGAP1 by means of specific siRNA transfection lead to inhibition of proliferation by 57.4±4.8% (p<0.0001) (g). Similarly, mitogen-induced CASMC migration was sharply inhibited by 48±9% in RanGAP1 siRNA transfected cells (p = 0.0001) (h). Concomitantly, the phenotype of siRNA RanGAP1 treated CASMC showed a significant difference in the cellular size index (length/width; 7.8±2.5 vs. 2.5±0.9 p = 0.002) (i), indicating a phenotypic change that is consistent with contractile, quiescent CASMC.

Cy3 labelled control siRNAs revealed a transfection efficacy of 94.5±4% in CASMC (data not shown). Treatment of CASMC with RanGAP1 siRNA reduced RanGAP1 protein by 31.8±21.2% (90 kD band) and 75±14.7% (70 kD band) 48 h post transfection, respectively (Figure 1B). The cyclin-dependent kinase inhibitor p27^Kip1^ was highly expressed in quiescent cells. Concomitantly, we observed a high level of p27^Kip1^ protein expression in RanGAP1 deficient cells (60±34%, Figure 1B). The LDH assay revealed no significant cytotoxicity indicating the absence of a potential cytotoxic effect that would be associated with RanGAP1 deficiency. Desmin, a marker of smooth muscle differentiation and cytoskeletal rearrangement, was found to be upregulated in quiescent cells as compared to proliferating cells [26]. RanGAP1 deficiency leads to an increase of desmin expression as depicted in Fig. 1B. Immunofluorescent examination of RanGAP1 in CASMCs transfected with control siRNA-SCR disclosed local expression of RanGAP1 at the nuclear rim and faint signals of cytosolic RanGAP1 (Figure 2 b–c). siRNA transfected CASMC underwent a readily visible change in cellular phenotype as assessed by light microscopy (Figure 2d).

Compared to control cells (Figure 2a), RanGAP1 deficient cells (Figure 2d) developed filiform extensions (>90% of cells), visualized by an increase in cell length (59±12 µm vs. 32±11 µm) and a decrease in cell width (8±2 µm vs. 13±1 µm), resulting in a higher length-to-width ratio compared to non RanGAP1 deficient cells (length/width; 7.8±2.5 vs. 2.5±0.9; p = 0.002). These morphometric changes are consistent with a synthetic CASMC phenotype [4].

siRNA mediated gene silencing of RanGAP1 caused a significant downregulation of cellular proliferation by 57.4±4.8% (p<0.0001), as determined by cell count (Fig. 2g). Furthermore, mitogen induced cellular migration was analyzed by a fibronectin gradient via a Boyden chamber assay. Migratory activity was significantly downregulated 18 h after RanGAP1 depletion of CASMC as compared to control cells (Fig. 2h).

### RanGAP1 expression is highly regulated during vascular lesion formation

**Figure 3:**
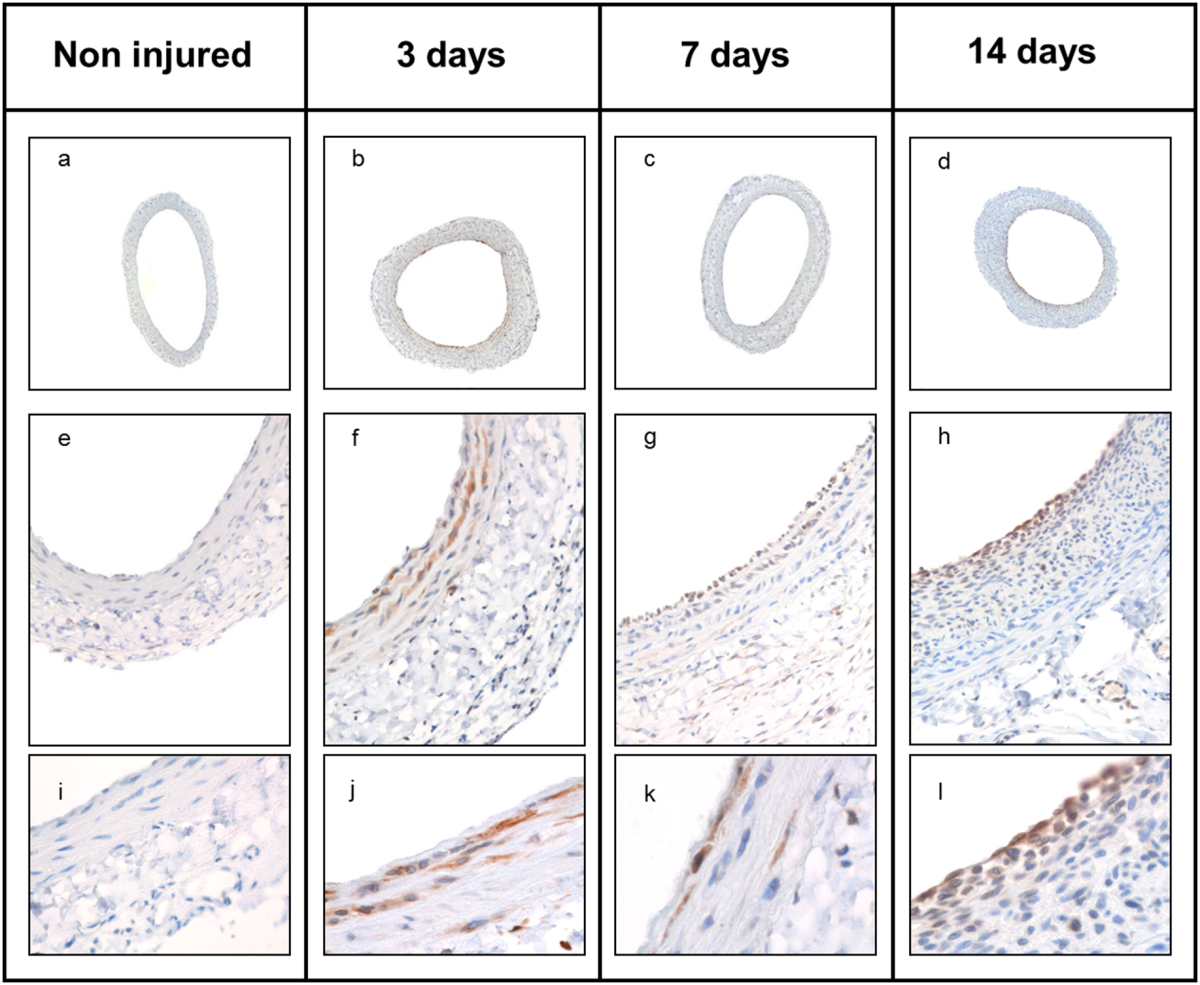
RanGAP1 expression in the rat carotid artery injury model. To determine the spatiotemporal expression pattern of RanGAP1 during the course of neointima formation, the rat carotid injury model was applied. Immunohistochemical staining revealed upregulation of RanGAP1 at day 3 (b, f, j, and day 7 (c, g, k) whereas RanGAP1 expression ceased when SMC proliferation decreases at day 14 (d, h, l) subsequent to balloon injury.

**Figure 4:**
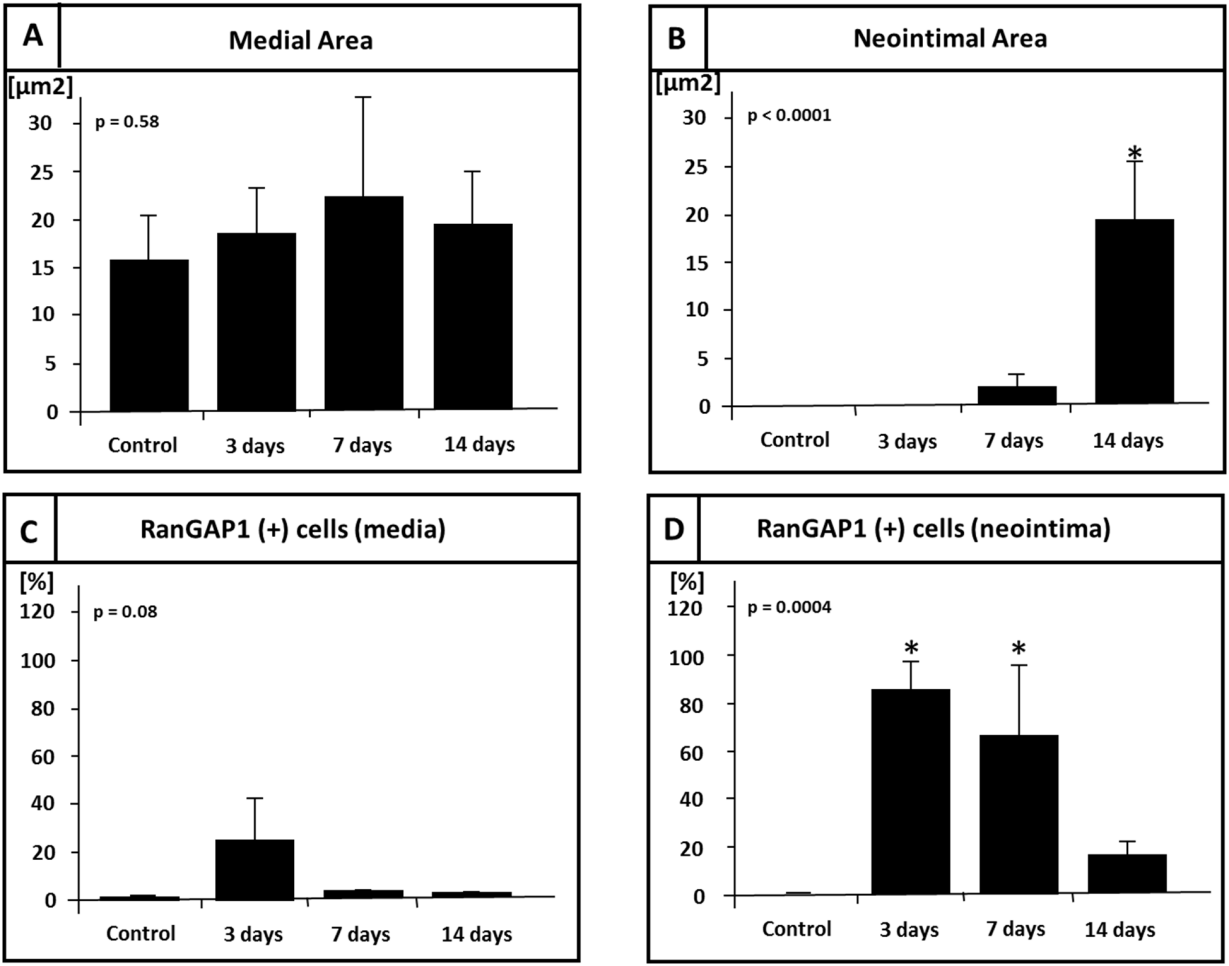
Quantitative morphometric and immunohistochemical analysis of neointima formation and RanGAP1 expression in the rat carotid artery injury model. No significant difference was detectable between the injured group compared to non-injured control arteries with respect to medial area at 3 days, 7 days and 14 days post injury (A) (p = 0.58). Of note, we observed in the media a trend towards a cellular upregulation of RanGAP1 (p = 0.08) 3 days after injury concomitantly with the beginning of cellular proliferation as a response to vascular injury. The increase of neointimal area was detectable at day 7 and peaked at 14 days following vascular injury (B) (p<0.0001). RanGAP1 expression in the media was the highest at the initiation of cellular proliferation and decreased to barely detectable levels at the completion of neointima formation (C). 3 days post injury, almost all cells in the neointima stained positive for RanGAP1 and subsequently, levels decrementally decreased at later time points, e.g. at day 7 and day 14 (D). In non-injured control sections, RanGAP1 expression was virtually undetectable.

The experiments above reveal that RanGAP1 is reduced as cells differentiate and suggest that this may be important for the process. To address the inverse question - is RanGAP1 induced as cells leave quiescence - we turned to the rat in vivo model of neointima formation. Here, we examined the spatiotemporal expression pattern of RanGAP1 in balloon-injured rat carotid arteries at 3, 7 and 14 days after balloon-mediated injury (Fig. 3). Sham operated animals served as control. Immunohistochemistry demonstrated that RanGAP1 levels are low in differentiated uninjured cells and that neointima formation is associated with a significant increase of RanGAP1 expression in the media and neointima at 3 and 7 days post injury within the neointima (Figure 4). Most likely as a reflection of SMC redifferentiation and abrogated proliferation at 14 days post injury, overall RanGAP1 expression decreased at the end of the process of neointima formation both in the media and neointima (Figure 4). Neointimal cells at the luminal surface showed persistent RanGAP1 expression, whereas deeper layers of the neointima showed almost absence of RanGAP1 expression. In contrast, RanGAP1 expression was barely detectable in uninjured control arteries.

## Discussion

Vascular smooth muscle cells are generally quiescent and thus proliferate at very low indices [20]. Vascular injury consistently induces CASMC dedifferentiation, migration and proliferation, thus forming the neointima. Concomitantly, CASMCs undergo phenotypical changes in response to mitogens such as growth factors and cytokines during this process [36].

**Figure 5:**
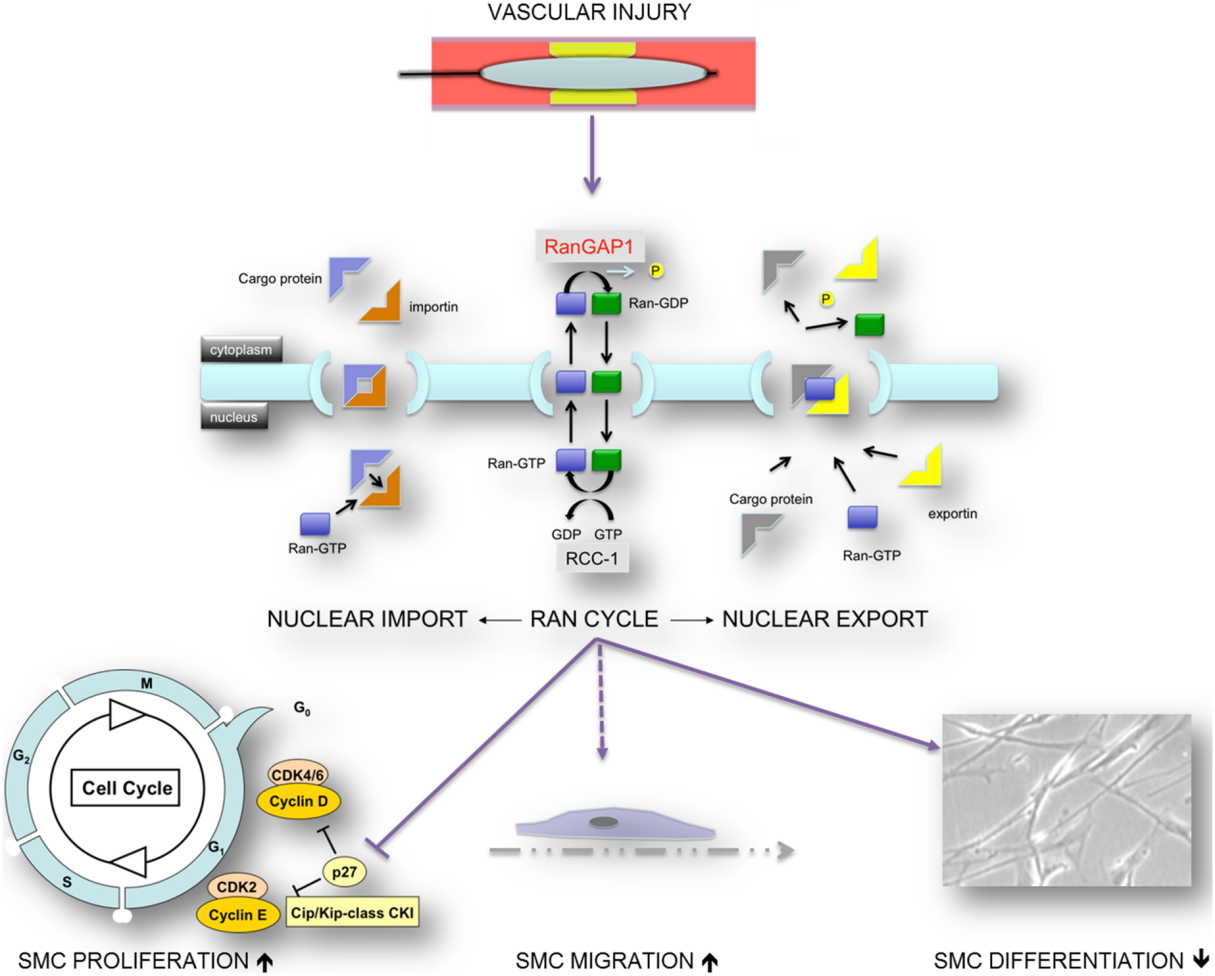
Model: RanGAP1, a key player in nucleocytoplasmic transport, plays a critical role in smooth muscle cell differentiation, migration and proliferation. Appropriate modulation of RanGAP1 expression may thus be a strategy to modulate vascular proliferative disease development such as restenosis.

Numerous factors have been implicated in driving differentiation and dedifferentiation processes in SMCs, including cell cycle proteins, transcription factors, classical mitogens as well as nucleocytoplasmic transport proteins [6], [35]. As mentioned previously, the latter includes specific nucleoporins and transport receptors. Here, we focused on the GTPase cycle of Ran, which is involved in nucleocytoplasmatic transport, spindle formation and nuclear envelope reformation [21]. We found that siRNA-mediated downregulation of the Ran regulatory protein RanGAP1 in CASMC correlates with a change of the cellular phenotype toward a differentiated state, inhibition of cellular proliferation and migration without evidence of cytotoxicity. Assessment by light microscopy revealed a change of the cellular phenotype of RanGAP1 deficient CASMCs into a spindle-like, contractile phenotype, which is suggestive of a non proliferative phenotype of CASMCs (Figure 5) [22], [25]. Concomitantly, Western blot assessment of RanGAP1 depleted cells demonstrated an upregulation of desmin, an established marker of a differentiated CASMC phenotype [23]. *In vivo*, we observed low levels of RanGAP1 expression in quiescent, differentiated CASMCs in uninjured rat carotid arteries and a strong expression of RanGAP1 in proliferating vascular cells in balloon-injured arteries, suggesting a linkage of RanGAP1 expression with cellular activation, dedifferentiation, proliferation and migration in the vascular wall following injury. Further, we could show in an established cell migration model that CASMCs dramatically decreased in their migratory capacity when they were deficient of RanGAP1.

What could be the underlying mechanisms? Nucleocytoplasmic transport is an essential process in all cells including differentiated vascular smooth muscle cells [34], and quantitative depletion of RanGAP1 is known to be incompatible with life. However, numerous transport pathways involving approximately 20 different transport receptors exist that have differential requirements for Ran GTP levels in cells [7]. It is hence conceivable that reduction of RanGAP1 levels selectively inhibits some but not all transport pathways. When cells re-enter the cell cycle, need for nuclear transport, e.g. import of histones in S-phase, dramatically increases [24]. Moreover, very high demands for an efficient Ran GTPase cycle may be needed specifically in mitosis, when Ran contributes to nuclear envelope breakdown and spindle formation [27].

Cellular dedifferentiation is one of the initial mechanisms that are triggered in the formation of neointima. To further investigate the relevance of RanGAP1 *in-vivo*, the rat carotid balloon-injured model was applied. Early after balloon mediated vascular injury, SMC dedifferentiate from a contractile to a synthetic phenotype [28]. In this phase, we observed a marked upregulation of RanGAP1 during the active phase of cellular proliferation and the process of neointima formation. Of note, neointimal cells located at the luminal surface revealed persistent RanGAP1 expression, as opposed to cells in deeper layers of the neointima where RanGAP1 expression was less or not detectable at all. This observation is in line with upregulation of cell cycle regulatory factors like cyclin E at the luminal surface 1–3 weeks after injury in another rat carotid artery study. [29] Vascular SMC proliferation requires a coordinated expression of cyclin-dependent kinases (CDKs) and its regulatory subunits (cyclins). The CDK activity is negatively regulated by the interaction with specific CDK inhibitory proteins (CKIs) like p27^Kip1^. [29], [30] We showed that cyclin-dependent kinase inhibitor p27^Kip1^ was highly expressed in quiescent cells and high levels of p27^Kip1^ protein expression in RanGAP1 deficient cells. This is in accordance with findings from a balloon angioplasty study in rat arteries showing the induction of p27^Kip1^ in vascular SMCs and the contribution of p27^Kip1^ upregulation in the re-establishment of the quiescent phenotype. [32]–[33]. In addition, overexpression of p27^Kip1^ leads to abrogation of vascular SMC migration [31], which was also a finding in our study subsequent to RanGAP1 depletion. Since downregulation of RanGAP1 interferes with proliferation and a dedifferentiated phenotype of CASMC, we hypothesize that conversely, inhibition or at least modulation of RanGAP1 expression may be sufficient to attenuate the vascular proliferative response following vascular injury, possibly even without an appreciable cytopathic effect. However, this remains to be shown in a subsequent experimental setting.

### Limitations

Targeting RanGAP1 expression may be sufficient to attenuate the vascular proliferative response following vascular injury, possibly even without an appreciable cytopathic effect. However, this remains to be accordingly shown in subsequent experiments. In addition, we have not investigated RanGAP1 in the setting of an atherosclerotic animal model. This may be of particular interest for further investigations since p27Kip1 showed an inversely correlated expression in atherosclerotic lesions [32]–[33].

In summary, we have shown that RanGAP1 protein levels are regulated during vascular proliferative processes and associated with SMC differentiation, migration and proliferation both in cell culture as well as in the intact animal. Our findings suggest that regulation of nucleocytoplasmatic transport processes, mitosis and cell cycling by RanGAP1 plays an intrinsic role for the phenotypical changes of CASMCs in the context of vascular proliferative diseases. Thus, it is tempting speculate yet not proven at this stage that targeting RanGAP1 may provide an interesting therapeutic tool to modulate vascular proliferative diseases such as neointima formation and thus restenosis after percutaneous intervention.
